# Emerging therapeutic targets in myeloproliferative neoplasms and peripheral T-cell leukemia and lymphomas

**DOI:** 10.1080/14728222.2018.1406924

**Published:** 2017-11-24

**Authors:** Anna Orlova, Bettina Wingelhofer, Heidi A. Neubauer, Barbara Maurer, Angelika Berger-Becvar, György Miklós Keserű, Patrick T. Gunning, Peter Valent, Richard Moriggl

**Affiliations:** ^a^ Institute of Animal Breeding and Genetics, University of Veterinary Medicine Vienna, Vienna, Austria; ^b^ Ludwig Boltzmann Institute for Cancer Research, Vienna, Austria; ^c^ Institute of Pharmacology and Toxicology, University of Veterinary Medicine Vienna, Vienna, Austria; ^d^ Medicinal Chemistry Research Group, Research Centre for Natural Sciences, Hungarian Academy of Sciences, Budapest, Hungary; ^e^ Department of Internal Medicine I, Division of Hematology and Hemostaseology, Medical University of Vienna, Vienna, Austria; ^f^ Ludwig Boltzmann-Cluster Oncology, Medical University of Vienna, Vienna, Austria; ^g^ Department of Chemical & Physical Sciences, University of Toronto Mississauga, Mississauga, Canada; ^h^ Department of Chemistry, University of Toronto, Toronto, Canada; ^i^ Medical University Vienna, Vienna, Austria

**Keywords:** Epigenetic target, hematopoietic cancer, MPN, mutational landscape, PTCL, therapeutic target

## Abstract

**Introduction**: Hematopoietic neoplasms are often driven by gain-of-function mutations of the JAK-STAT pathway together with mutations in chromatin remodeling and DNA damage control pathways. The interconnection between the JAK-STAT pathway, epigenetic regulation or DNA damage control is still poorly understood in cancer cell biology.

**Areas covered**: Here, we focus on a broader description of mutational insights into myeloproliferative neoplasms and peripheral T-cell leukemia and lymphomas, since sequencing efforts have identified similar combinations of driver mutations in these diseases covering different lineages. We summarize how these pathways might be interconnected in normal or cancer cells, which have lost differentiation capacity and drive oncogene transcription.

**Expert opinion**: Due to similarities in driver mutations including epigenetic enzymes, JAK-STAT pathway activation and mutated checkpoint control through TP53, we hypothesize that similar therapeutic approaches could be of benefit in these diseases. We give an overview of how driver mutations in these malignancies contribute to hematopoietic cancer initiation or progression, and how these pathways can be targeted with currently available tools.

## 1. Introduction

Hematopoietic malignancies arise when somatic hematopoietic stem cells (HSCs) or lymphoid or myeloid progenitor cells acquire driver mutations that change cellular differentiation fates and overcome senescence. During leukemic development, healthy hematopoietic cells switch from normal to enhanced transcription by increasing the number of super- or stretched-enhancers at promoter or enhancer elements. Subsequently, new chromatin loop structures form that change topologically associated domains, which trigger reprogramming of cancer cells [1,2]. We are starting to gain insights into organized chromatin regulatory circuits that not only contain proteins and DNA, but also structural or regulatory RNA, to promote oncogenic gene transcription [3].

During the evolution of hematopoietic diseases, physiologic polyclonal hematopoiesis switches to abnormal monoclonal or oligoclonal hematopoiesis, which involves an increased response to cytokine signaling that is often associated with mutated tyrosine kinases (TKs) and GTPases. Despite advances in understanding the pathophysiology of hematopoietic diseases, developing new therapeutics for patients remains challenging. Not all mutated key genes are ‘easy targets’ and patients still frequently relapse. However, we understand that increased oncogene transcription and silenced tumor suppressor genes facilitate neoplastic growth, survival, and clonal expansion. Furthermore, changes in the epigenome result in abnormal transcription factor/cofactor/corepressor networks at promoter–enhancer interactions. Here, we provide an overview on novel insights and novel targeting approaches against drivers of myeloproliferative neoplasms (MPNs), secondary acute myeloid leukemia (sAML), and peripheral T-cell leukemia and lymphomas (PTCLs). We also discuss the feasibility of targeting new players as the focus of new therapeutic developments.

## 2. Identification of key driver mutations in hematopoietic cancer

The last decade of cancer research has been significantly shaped by major advances in next-generation sequencing technologies that have led to the analysis of more than 100,000 whole cancer genomes [4]. Approximately 4 million coding mutations have been identified, defining 500 genes that act as functional cancer drivers [5,6], many of which participate in core cancer pathways [7]. The ability to define hyperactivated signaling pathways that are commonly deregulated allows for development of new therapies. One strategy focuses on the development of drugs designed to directly target driver oncoproteins that cancer cells are addicted to. In contrast, specific targeting of a tumor suppressor to restore normal function has been reported but remains challenging. However, loss of tumor suppressors, such as SOCS2 or PRC2, leads to hyperactivation of the JAK-STAT pathway which could be targeted instead [8,9].

We follow the current paradigm of somatic mutation theory in cancer development, which is based on clonal expansion upon the occurrence of mutations rather than mutations being a consequence of cancer itself. Current cancer drug development is primarily focused on the finding and targeting of driver mutations. However, evidence of both mutation-free tumors and the presence of driver mutations in healthy patients (discussed in more detail in the TP53 section), may support alternative theories. For example, epigenetic gene regulation is tightly linked with metabolism, steered by complex cytokine, adipokine, growth factor, and/or hormone signaling, which can also influence cancer cells. It is known that cancer cells may harbor a variety of somatic alterations in various biological pathways, but only part of these mutations are related to cancer initiation and development and only a few are driving the cancer progression. Accordingly, upon treatment with a single targeted drug, it is often difficult to predict the outcome because of the compensatory pathways and feedback loops that are still poorly understood in most cancers [10–12].

The landmark demonstration of targeting a driver oncogene product was the development of Imatinib to inhibit BCR-ABL1 in chronic myeloid leukemia (CML). This led to a dramatic improvement in CML patient survival. It is therefore not surprising that multiple small molecule inhibitors were developed to target a range of TKs with remarkable clinical success. However, despite the exceptional activity of Imatinib in CML, drug resistance develops rather frequently in these patients. This clinically challenging condition is often associated with further genetic aberrations in the driver itself (BCR-ABL1) or with mutations in other critical target genes. Moreover, epigenetic and other mechanisms may promote upregulation of the STAT3/5 pathway, allowing cancer cells to escape drug action [8]. Combining different therapies against multiple ‘oncogene addictions’ could be a possibility to overcome primary or acquired resistance. Targeted approaches in solid cancers inhibit common downstream mediators of known oncogenes, such as MEK-ERK or the PI3K-AKT-mTOR kinase pathways, and similar pathways may also play a role in oncogenesis in hematopoietic malignancies [13]. Last, newer therapeutic strategies aim to exploit the specific dependency of cancer cells on basic cellular processes such as cell division, chromatin regulation, and metabolism.

**Figure 1.**
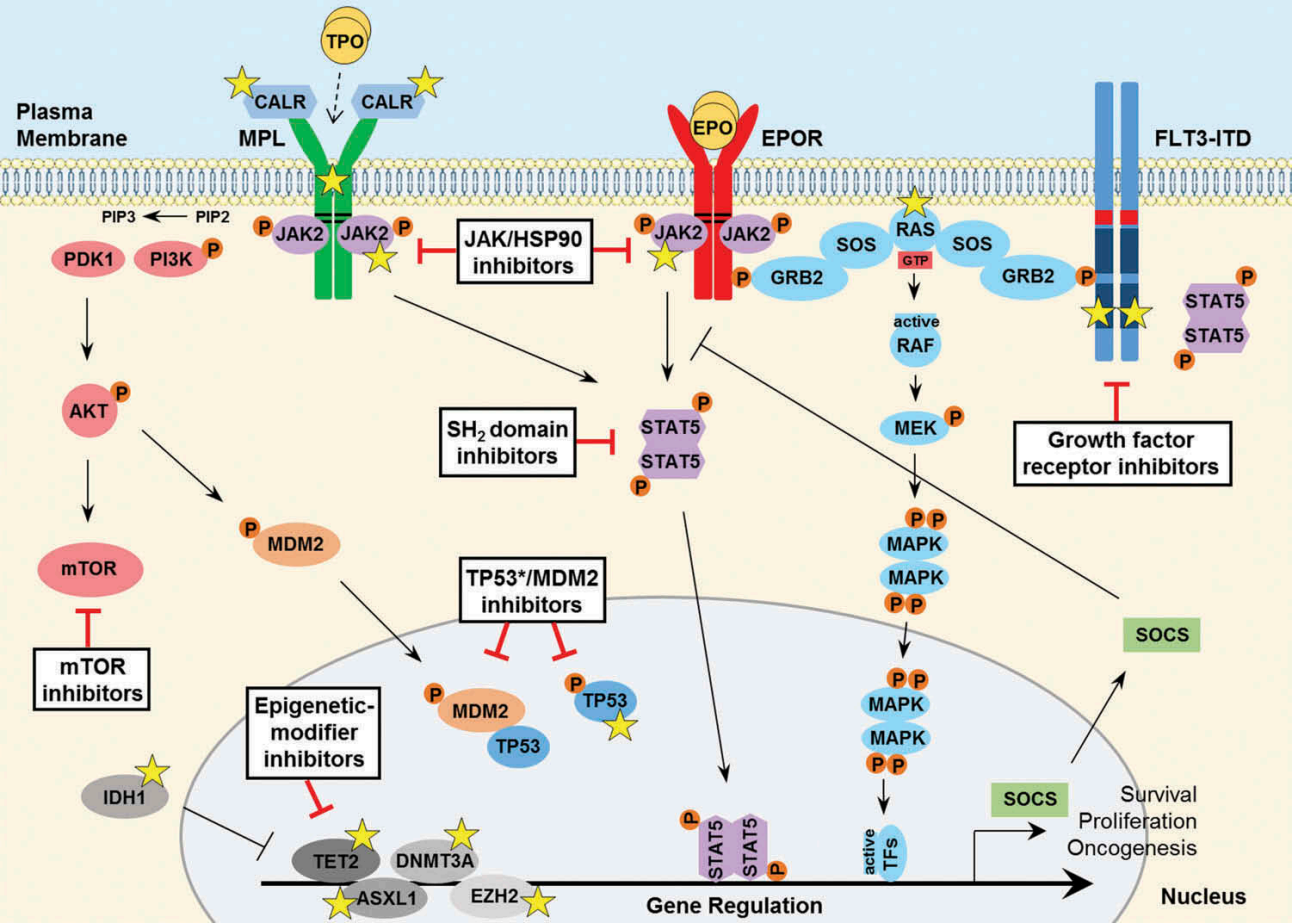
**Signaling pathways involved in the pathogenesis of MPNs and secondary AML**. JAK2 binds to the cytosolic juxta-membrane region of dimeric cytokine receptors such as MPL (TPOR) and EPOR, via the BOX1 and BOX2 receptor motifs (black lines). JAK2 activation (via receptor-ligand binding or gain-of-function mutation such as JAK2 V617F) promotes various downstream signaling pathways, via STAT5, including RAS-MAPK and PI3K-AKT. These pathways facilitate oncogenic gene transcription and promote cancer cell survival, proliferation or migration. The expression of negative regulators such as the SOCS proteins are induced by the JAK-STAT pathway, however they are not sufficient to block hyperactive JAK-STAT signaling and cannot bind JAK2 V617F. The FLT3-ITD mutant growth factor receptor commonly found in AML patients signals independently of ligand-binding, as a result of the internal tandem duplication (ITD) found within the juxta-membrane domain (red box) and point mutations that occur within the kinase domain (most frequently at D835; dark blue box) of the FLT3 receptor. FLT3-ITD hyperactivation promotes RAS-MAPK, PI3K-AKT as well as STAT5 signaling. A number of important somatic mutations have been reported in various oncogenes and tumor suppressor proteins within these pathways (yellow stars), where such mutations are known to contribute to disease initiation and progression. For further details on these mutations, see Table 1. Mutated calreticulin (CALR), frequently found in MPN patients, interacts with the extracellular portion of the MPL receptor at the Endoplasmic Reticulum-Golgi apparatus and also at the cell surface, promoting direct dimerization, activation of JAK2 and downstream signaling, independently of TPO binding (which is required for normal MPL signaling, indicated by a dashed arrow). Loss-of-function mutations in the critical tumor suppressor protein TP53 are also reported generally in MPN patients that progress to secondary AML. Furthermore, various epigenetic-modifier proteins are found to be mutated in MPN patients, including isocitrate dehydrogenase 1 (IDH1), methylcytosine dioxygenase TET2, DNA methyltransferase 3A (DNMT3A), Polycomb group protein ASXL1 and the histone methyltransferase protein of polycomb repressive complex 2 (PRC2) EZH2. Promising therapeutic agents to target these key proteins/pathways in MPN/AML have been developed and are summarized here (black boxes). TPO, thrombopoietin; EPO, erythropoietin; GTP, guanosine triphosphate; TF, transcription factor.

**Figure 2.**
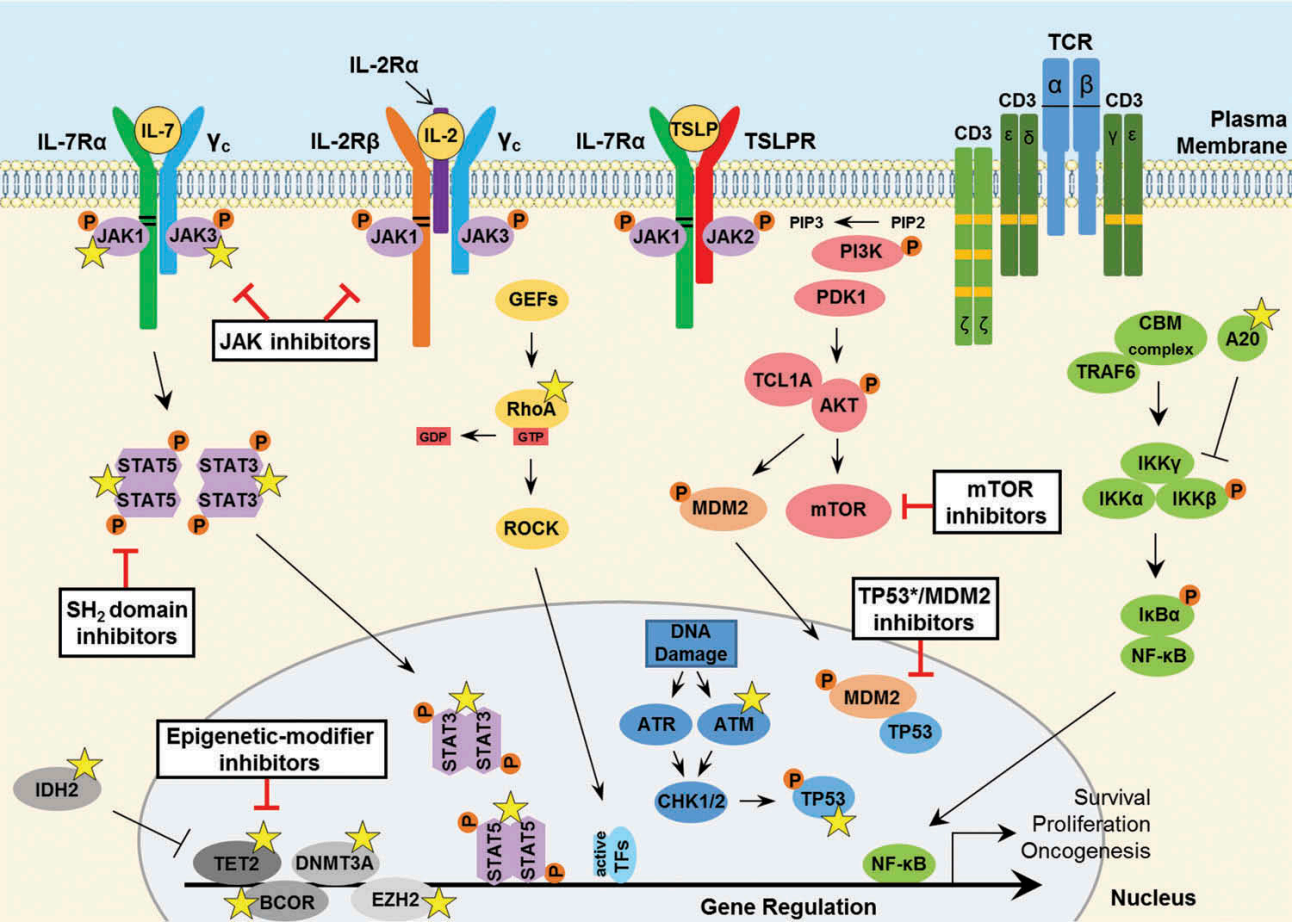
**Signaling pathways involved in the pathogenesis of PTCL**. JAK tyrosine kinases bind to the cytosolic juxta-membrane region of dimeric T-cell cytokine receptors such as IL-7Rα, IL-2Rβ, TSLPR and the common gamma chain (γ_c_). Conserved juxta-membrane BOX1 and BOX2 cytokine receptor motifs known to bind JAKs are indicated with black lines. Cytokine receptor-ligand binding promotes STAT3/5 tyrosine phosphorylation to facilitate gene transcription to promote cancer cell survival, proliferation or migration. A number of important somatic mutations have been reported in various oncogenes and tumor suppressor proteins within these pathways (yellow stars), where such mutations are known to contribute to disease initiation and progression. For further details on these mutations, see Table 2. GTPase signaling through RAS-RAF (not shown) or mutated RhoA-ROCK pathways are frequently activated in PTCL. T-cell receptor (TCR) activation, involving phosphorylation of immunoreceptor tyrosine-based activation motifs (ITAMs; orange boxes), triggers various downstream pathways including PI3K-AKT and NF-κB signaling. Furthermore, overexpression of the AKT-activating protein TCL1A, resulting from rearrangements between a TCL1 family gene and TCR loci rendering it under the control of TCR expression-regulating elements, can contribute to aberrant survival signaling and enhanced TCR activation. Loss-of-function mutations in the critical tumor suppressor proteins TP53 and ATM are reported in PTCL. Moreover, various epigenetic-modifier proteins are found to be mutated in PTCL patients, including isocitrate dehydrogenase 2 (IDH2), methylcytosine dioxygenase TET2, DNA methyltransferase 3A (DNMT3A), BCL-6 corepressor (BCOR) and the histone methyltransferase protein of polycomb repressive complex 2 (PRC2) EZH2. Promising therapeutic agents to target these key proteins/pathways in PTCL have been developed and are summarized here (black boxes). IL, interleukin; TSLP, thymic stromal lymphopoietin; CBM, CARMA3-BCL10-MALT1; GEF, guanine nucleotide exchange factor; GTP, guanosine triphosphate; GDP, guanosine diphosphate; TF, transcription factor.

We will first describe key genetic drivers in MPN (Figure 1) and subsequently discuss driver events in PTCL (Figure 2).

**Table 1.** Mutational landscape of myelofibrosis (MF), essential thrombocytopenia (ET), and polycythemia vera (PV).

Ref	Gene	Function	Frequency %		
			MF	ET	PV
[30]	*JAK2*	Tyrosine kinase	55–60	50–60	95–97
[31]	*CALR*	Endoplasmic chaperone	25–30	20–25	<1
[32]	*MPL*	Growth factor receptor	5–10	3–5	<1
[30]	*SF3B1*	Splicing regulator	5–10	~1	~1
[33]	*TP53*	DNA damage response	2–4	<1	<1
[34]	*CBL*	E3 ubiquitin ligase	5–10	0–2	Rare
[35]	*DNMT3A*	DNA methyltransferase	5–12	1–5	5–10
[35]	*TET2*	Methylcytosine dioxygenase	10–20	5	10–20
[36]	*EZH2*	Chromatin regulator	5–10	~2	~2
[37]	*IDH1/2*	Isocitrate dehydrogenase	3–5	<1	~2
[37]	*ASXL1*	Chromatin regulator	15–35	5–10	2–7

**Table 2.** Mutational landscape of angioimmunoblastic T-cell lymphoma (AITL) and peripheral T-cell lymphoma not otherwise specified (PTCL-NOS).

	Frequency %							
	*RHOA*		*TET2*		*DNMT3A*		*IDH2*	
Ref	AITL	PTCL-NOS	AITL	PTCL-NOS	AITL	PTCL-NOS	AITL	PTCL-NOS
[93]	53.3	7.7	nd	nd	nd	nd	nd	nd
[94]	67	18	73	29	23	12	13	0
[95]	71	17	82.6	48.5	26	27.3	30.5	0
[65]	71.8	27	59	46	38.5	36.6	33	4

## 3. Targets in MPN and driver mutations

MPNs are characterized by enhanced proliferation and reduced differentiation and cell death in one myeloid lineage, leading to the outgrowth of a dominant myeloid cell type in addition to extramedullary hematopoiesis. The World Health Organization (WHO) classification recognizes three main variants of BCR-ABL1-negative MPN: essential thrombocythemia (ET), polycythemia vera (PV), and myelofibrosis (MF) [14]. They are defined by excessive production of leukocytes, platelets, and/or erythrocytes in the bone marrow as well as by extramedullary myelopoiesis [14]. In MF patients, bone marrow fibrosis is also seen [14]. In the initial (chronic) phase of MPN, cellular differentiation and maturation is largely preserved and the expanded cell populations are functionally intact [14]. Apart from severe thromboembolic events, transformation to sAML is the most devastating complication experienced by MPN patients. AML evolution is seen in ~20% of patients with MF, ~5% of PV, and ~1% of ET patients [15–18]. During the early stage of MPN, clonal cells are usually responsive to hydroxyurea (HU), interferon-alpha (IFN-A), and/or Ruxolitinib, but this is not the case in most patients with advanced MPN or sAML. Several new clinical trials in advanced MPN focus on combination trials using various targeted drugs, including JAK2 TK inhibitors (TKI).

The main mutations that are currently used as clinically relevant diagnostic markers are driver mutations in *JAK2, CALR,* and *MPL* [14]. We describe below recurrent hotspot mutations in MPN, in key genes that constitute core cancer pathways.

### 3.1. JAK2 *point mutations or exon 12 mutations*

The *JAK2 V617F* mutation is present in 95% of PV, ~60% of ET, and ~45% of MF patients [15,19]. Surprisingly, the *JAK2 V617F* mutation has no clear association with survival or sAML transformation. The second most frequent mutation in *JAK2* occurs in exon 12 with a small deletion causing similar functional consequences as JAK2 V617F. This deletion occurs in a small percentage of JAK2 V617F-negative PV patients, but not in ET or MF. JAK2 activates STAT3/5A/5B transcription factors, which can directly induce target genes to accelerate cell cycle progression, survival, and cancer cell metabolism. It was shown through genetic experiments that particularly the activation of the two STAT5 transcription factors is crucial for PV [20]. Hyperactive JAK2 promotes prominent activation of the PI3K-AKT-mTOR and the RAS-RAF/MAPK-ERK pathways, among other less prominent signaling pathways, and evades negative-regulation by SOCS proteins [13].

JAK2 may be involved in directly or indirectly reprogramming epigenetic gene regulation; however, this is still controversial [21]. JAK2 is known to phosphorylate histone H3, thereby disrupting the binding of heterochromatin protein 1 alpha (HP1α) to chromatin [21,22]. Furthermore, JAK2 phosphorylates the arginine methyltransferase PRMT5, impairing its ability to methylate histone substrates, ultimately driving myeloproliferation [22].

### 3.2. CALR *exon 9 mutations*

The *CALRETICULIN* (*CALR*) driver mutation was identified in approximately 73% of *JAK2/MPL* mutation-negative ET and MF patients [23]. Mutations occur in exon 9 of *CALR* in the majority of *JAK2* wild-type MPN cases. CALR constitutes a key component of the quality-control machinery that ensures proper glycoprotein folding and Ca^2+^ homeostasis. In MPN, mutant CALR interacts with the thrombopoietin receptor (MPL/TPOR) promoting direct dimerization and activation of JAK2 at the endoplasmic reticulum (ER)–Golgi apparatus. The capacity of CALR to bind Ca^2+^ and regulate its homeostasis is lost due to a frame shift mutation in the carboxy-terminal Ca^2+^-binding domain [24]. Surprisingly, a functional cytokine-TK-STAT signaling hub at the cell membrane seems to be dispensable in CALR-mutated cells. Interestingly, and very reminiscent, STAT5 activation at the ER–Golgi was also described in Flt3-ITD^+^ or KIT D816V^+^ AML cases. Analysis of patient data suggests that *CALR* mutation-positive patients have a more favorable clinical outcome than patients with *JAK2* or *MPL* mutation-positive MPNs due to a lower risk of thrombosis [23].

### 3.3. MPL/TPOR *point mutations*

Somatic mutations affecting *MPL* are seen in up to 15% of *JAK2 V617F*-negative ET and MF patients. The most common gain-of-function mutation W515L leads to hyperphosphorylation of JAK2, STAT3, STAT5, ERK, and AKT proteins [25–27].

### 3.4. Chromatin remodeler mutations in MPN

Emerging evidence suggests that MPNs are likely the result of combined genetic deregulation of several mutated genes encoding for epigenetic regulators. Mutations in epigenetic remodelers have been described for *TET2* (12%), *ASXL1* (5%), *DNMT3A* (5%), *EZH2* (~3%), and *IDH1* (~1.5%) [28]. All of these epigenetic modifiers act either on DNA or histone/transcription factor methylation. Interestingly, they appear to be the most frequent somatic mutations after *JAK2* and *CALR* in MPN [29]. However, these mutations are not restricted to MPN and are also found in a wide spectrum of other neoplasms, including AML. It is thought that the development of clonal evolution in MPN is slow and often includes a clinically ‘silent’ phase. As a result, most mutations are already present at diagnosis. Interestingly, the order in which mutations are acquired may play an important role in the development of the disease phenotype. The reversible nature of epigenetic changes may make them good potential therapeutic targets. An overview of the described mutations as well as other relevant mutations not mentioned here is shown in Table 1.

### 3.5. TP53 *point mutations in MPN and secondary AML*

TP53 senses DNA damage and mitotic checkpoint control, and mutations in the *TP53* gene (TP53*) are most frequent in patients with sAML. The *TP53* mutations are represented by bi-allelic or homozygous mutations [38]. Interestingly, TP53* heterozygosity is detected in MPNs, but homozygous or compound mutations are only detected in sAML [28]. Notably, loss-of-function mutations in *TP53* appear to emerge during disease progression. It is currently under discussion whether cytoreduction upon HU therapy selects for TP53* mutated cells. A recent study analyzed the impact of TP53* in MPN patients and, although it is common that at least one somatic TP53* allele is transcribed in patient cells, the authors did not find a direct association between TP53 inactivation and HU resistance or blast transformation [33]. TP53 can also interact with STAT3 and STAT5 [39,40] and it induces mRNA expression of *STAT5A*, but not of *STAT5B* [41]. Overall, current sequencing data suggest that the age of patients is the strongest factor affecting low-burden TP53* incidence in MPN, which may persist for years without an immediate risk of progression.

### 3.6. GTPase *gain-of-function mutations*

*GTPases* are among the most frequently mutated genes in cancer. The amino acid sequences of the entire family are highly conserved making their targeting very difficult. Although infrequent in MPN, more than 10% of AML cases harbor activating *RAS* mutations. RAS-RAF signaling can be triggered by normal cytokine, growth factor, or hyperactive JAK action and it constitutes a core cancer pathway [7]. RAS-RAF is upstream of MAPK-ERK signaling and it can further activate RHO and RAC GTPase proteins, which were reported to be essential for nuclear shuttling of STAT5A [42]. Furthermore, oncogenic RAS requires mitochondrial functions of STAT3, illustrating that the RAS-RAF pathway is interconnected with aberrant JAK-STAT, cytokine, or growth factor signaling. Similarities in the GTPase superfamily and their pleiotropic biological functions have made targeting attempts in clonal myeloid diseases unsuccessful to date [43].

## 4. Targets in PTCL and driver mutations

PTCLs represent a heterogeneous mature T-cell disease group of 15% of all non-Hodgkin lymphomas, which are often accompanied by aggressive organ infiltrations. PTCL can be variable in terms of immunophenotypic, morphological, and molecular features [44–46]. The 2016 WHO classification of lymphoid neoplasms distinguishes more than 20 mature T- and natural killer-cell neoplasms [47]. Within this rather rare disease group, the most common subtype is PTCL-not otherwise specified (NOS), which summarizes cases not attributable to other entities, followed by angioimmunoblastic T-cell lymphoma (AITL), ALK^+^ anaplastic large cell lymphoma (ALCL), and ALK^−^ ALCL [46,48,49]. Further subtypes include adult T-cell lymphoma/leukemia (ATLL), T-cell prolymphocytic leukemia (T-PLL), and cutaneous T-cell lymphoma (CTCL) including mycosis fungoides and Sézary syndrome (SS) [50,51]. The only frequent recurring chromosomal translocation identified in the PTCLs is the t(2;5)(p23;q35) NPM-ALK fusion characteristic of ALK^+^ ALCL. So far, no other genetic alterations (e.g. ITK-SYK translocation [52], IRF4 rearrangements [53], abnormalities, or deletions in chromosome 6q, 7q, or 9q [54–56]) have been linked to diagnosis, complicating clinical decisions in the treatment of PTCL patients. Moreover, combined chemotherapy (CHOP; Cyclophosphamide, Doxorubicin, Vincristine, Prednisolone; or CHOEP with Etoposide) often results initially in favorable response rates, however relapses and refractory disease are frequently observed [45,57]. Stem cell transplantations can only be offered to certain patients, and it can therefore be speculated that beneficial outcomes after transplantation may be attributed to patient selection [58]. So far, there is no optimal therapeutic approach to treating PTCL patients and the identification of molecular targets is of great importance.

High-throughput methodologies were used to identify the cell of origin and to characterize commonly altered pathways in PTCL [51,59–67]. These efforts were successful in defining characteristic gene expression profiles for AITL, ALK^+^ ALCL, ATLL, and PTCL-NOS, identifying recurrent mutations and recognizing specific subtypes, which can now help to support correct diagnosis and classification of patients to distinct disease subgroups with different prognoses [46,59,60,62,63].

PTCL patient numbers in sequencing studies are limited, but certain classes of genes and pathways are commonly affected in a majority of cases. The emerging understanding of mutations involves signaling pathways and epigenetic reprogramming, which highlight new targeting concepts. This is an intensive research area and many new reagents have been developed to define novel combinatorial treatments [68]. In the following we focus on PTCL, but we exclude detailed descriptions for ALK^+^ and ALK^−^ ALCL, since this has been extensively reviewed elsewhere [69].

### 4.1. TCR activating mutations

Mutations constitutively activating TCR signaling are most frequent in T-PLL, where the *TCL1A* inversions/translocations upstream of mTOR signaling rearrange with the *TCR* locus [70]. Enhanced TCL1A expression in T-PLL also amplifies TCR signaling. Moreover, TCR activation was linked to PTCL-NOS with the ITK-SYK fusion gene being present in approximately 10% of cases [52,71], or *MYC* overexpression due to IRF4 activating fusions [72] mimicking survival signals normally emanating from antigen receptor signaling. Furthermore, missense mutations in *TNFAIP3*, encoding the negative regulator of NF-κB activation A20 in T-cells after TCR stimulation [73], mutations in WNT/β-Catenin negative regulators *APC* and *CHD8*, and other genes with known suppressive roles in TCR activation were disease associated [74]. *GATA3* and *TBX21* expression are both important in T-cell development, and mutations in these genes may be associated with the PTCL-NOS subgroups, representing potential diagnostic predictors and possibly also therapeutic targets [59].

### 4.2. Gain-of-function JAK/STAT pathway mutations

Reports on patient mutation sequencing analyses of various T-cell lymphoma subtypes frequently include the JAK/STAT pathway, where *JAK1, JAK3, STAT3*, and *STAT5B* are predominantly mutated to cause hyperactivation [64,70,75–78]. Importantly, ALK^−^ ALCL is associated with STAT3 activation [64] and recurrent, somatic activating mutations in the closely related *STAT5B* gene were reported. The *STAT5B N642H* mutation occurs with the highest frequency in PTCL, whereby most mutations cluster in the SH_2_ and C-terminal transactivation domain [70,75,78–82]. A recent study found that ~70% of T-PLL patients carry JAK-STAT hyperactivating mutations [70]. Deep sequencing of known recurrent somatic mutations in T-PLL revealed a mutational burden of 4% in *IL2RG*, the JAK3 binding receptor chain that is shared by interleukins that use the common gamma chain. *JAK1* is less frequently mutated (10%) compared with *JAK3* (30%) and *STAT5B* (36%) [70]. Indeed, studies have linked JAK3 and STAT5B mutations with poorer patient survival [83,84]. STAT5 activation was also linked to an autocrine PDGF signaling loop in PTCL-NOS [85]. Enhanced STAT5 signaling was also linked to overexpression of oncogenic miR-155 in CTCL [86], associated with downregulation of tumor-suppressive miR-22 [87], or enhanced disease progression caused by Lymphotoxin-α-dependent lymphangiogenesis [88]. STAT5-dependent CD80 expression was also linked to resistance to Vorinostat and risk of disease progression in PTCL [84,89,90].

### 4.3. Chromatin remodeler mutations in PTCL

Like in other hematopoietic neoplasms [91], *TET2, DNMT3A*, and *IDH2* mutations occur frequently across PTCL subtypes, although certain mutations seem to be confined to T-cell lymphoma cases [92] (see Table 2 for overview).

*TET2* frameshift and nonsense mutations were frequently identified in AITL (~70%), PTCL-NOS (~60% in T_FH_ cell marker expressing subtype), and CTCL (~10%) [75,96–98]. In AITL, *IDH2* and *TET2* mutations were detected in the same patients, which is not the case in myeloid malignancies [77]. In AITL and PTCL-NOS, *TET2* mutations were associated with a worse prognosis [96]. *Tet2*-deficient mouse models elicit altered T-cell differentiation and can develop T-cell lymphoma with T_FH_-like features [99,100]. In *Tet2*-knockdown mice, the outgrowth of T_FH_-like tumor cells was connected to methylation changes of *BCL-6* [100], the locus repressor of *STAT5A/B*.

Cancer genome sequencing efforts identified *DNMT3A* as one of the most frequently mutated genes in hematological malignancies, which raises the question of how these lesions promote malignant cell growth. DNMT3A functions as a *de novo* DNA methylation enzyme, but it also interacts with histone modifiers promoting gene repression [101,102] in cooperation with STAT5 [103] and EZH2 [104]. In AITL, PTCL-NOS and CTCL subtypes, *DNMT3A* mutations cluster in the methyltransferase domain. Interestingly, only about 20% of these mutations are at position R882 [75,77,94,95,97], the variant commonly found in myeloid diseases acting as a negatively regulating hypomorphic protein [105]. *Dnm3a*-deficient mice develop a PTCL-like disease at a frequency of 12% and heterozygous animals at a rate of 10%, associated with hypomethylation and decreased TP53 activity [106]. *TET2* and *DNMT3A* mutations likely occur early during evolution of hematopoietic neoplasms and are even detectable in apparently healthy individuals [99,107]. These mutations can also co-occur which emphasizes the importance of disrupted DNA and histone methylation in PTCL [77].

IDH2, normally catalyzing the conversion of isocitrate to alpha-ketoglutarate in the Krebs cycle, is frequently mutated in hematopoietic neoplasms resulting in novel enzymatic activity producing 2-hydroxyglutarate (2-HG). This oncometabolite represses H3K and DNA 5-mC demethylation by inhibiting TET2, thus leading to abnormal regulation of gene transcription which potentially promotes lymphomagenesis. This is underpinned by the finding that about 30% of AITL cases possess *IDH2* mutations [65,108]. Furthermore, TCR signaling and T-cell differentiation promoting genes are hypermethylated [65]. Interestingly, no *IDH1* mutations have been mapped in AITL as yet, and only the *IDH2 R172* mutant but not *IDH*2 *R140* (a frequent mutation in myeloid neoplasms) has been documented. An explanation may be given by murine knock-in models of the common IDH2 variants, which identified IDH2 mutated at R172 to produce the highest 2-HG levels in T-cells, thereby impairing lymphopoiesis [109]. Interestingly, AITL cases with *IDH2 R172* mutations show a distinct gene expression signature with downregulated T_H2_ differentiation genes (e.g. IFNG and STAT1) and upregulated IL-12 target genes [109]. In addition, mutations in epigenetic regulators catalyzing methylation and acetylation changes such as *EZH2, TET2*, and *BCOR* were found in a number of T-PLL patients [76].

### 4.4. TP53 *and diminished DNA damage response pathways*

*TP53* mutations were found in 14% of cases categorized as T-PLL [83]. However, overexpression and accumulation of wild-type TP53 is common in T-PLL [110]. In ALK^+^ ALCL, which is driven by NPM-ALK, the fusion kinase can efficiently block wild-type TP53 function [111]. Still, in other PTCL diseases *TP53* mutations are not as frequent as in MPN. In addition, patient age could bias the analysis of *TP53* mutations.

The *ataxia telangiectasia mutated* (*ATM)* tumor suppressor frequently displays loss-of-function mutations or is deleted in PTCL [70]. ATM is centrally involved, in conjunction with ATR and CHK2, in activation of the DNA damage checkpoint control, and it maintains the balance between thymocyte survival and apoptosis, especially during V(D)J recombination [112]. DNA double-strand breaks result in rapid activation of ATM/ATR, in turn activating substrates that regulate cell-cycle progression, DNA repair, and cell death. Interestingly, ATM is also known to interact with TCL1 which was described to result in enhanced NF-κB activity and cell proliferation in association with TCR signaling in PTCL. In addition to immune defects, ataxia-telangiectasia (AT) patients and *Atm^–/–^* mice share a predisposition to T-cell malignancies, pointing toward a common etiology for these two consequences of ATM inactivation. The risk of developing a lymphoid neoplasm is increased approximately 200-fold in AT patients compared with the normal population. The frequency of T-cell tumors in AT patients exceeds that of B-cell malignancies by fourfold, and myeloid cancers have yet to be reported. The vast majority of lymphoid tumors that develop in children with AT are T-cell ALL/lymphoma, while young adults are mostly predisposed to T-PLL [112].

In this context, it is of mechanistic interest to link JAK and STAT3/5 activation to regulation of reactive oxygen species (ROS) generation, which is known to cause DNA damage, promoting mutations, oxidization of lipids, or silencing of phosphatases by catalytic cysteine oxidization [113–116]. Surprisingly, wild-type JAK2 decreases detoxifying glutathione S-transferases in epithelial cells, enhancing oxidative damage. In contrast, expression of anti-oxidative scavengers are under the control of STAT5, illustrating the interplay between JAK2 and STAT5 in balancing ROS action [117,118]. RAD51 members are conserved down to the *E. coli* RecA proteins and they are essential for DNA repair, which is downstream of cytokine- or TK-STAT signaling in mammalian cells [119,120]. However, the link between a hyperactive JAK-STAT pathway, TP53*, and mutated *ATM/CHK2* is poorly understood. These core cancer pathways need further characterization in order to understand drug actions, to overcome resistance mechanisms, and to finally eradicate cancer (stem) cells.

### 4.5. GTPase *gain-of-function mutations*

*RHOA* mutations are the only frequent GTPase mutations described in PTCL, occurring predominantly in up to 70% of AITL patients as well as 20% of PTCL-NOS and 15% of ATLL cases [93–95,121]. RHOA is a member of the Rho family of small GTPases that links cell-surface receptors to different intracellular signaling proteins. In its active GTP-bound state, RHOA functions in controlling the actin cytoskeleton and stress fibers [122]. The most prominent mutation is *RHOA G17V* which acts as a dominant-negative molecule, underpinning its tumor-suppressive function in T-cells [93–95]. Other mutations found frequently in ATLL were mostly located within the GTP-binding pocket, with the gain-of-function variant C16R as the most recurrent. However, loss-of-function mutations have also been detected [121]. Of note, in AITL and related lymphomas, *RHOA* mutations were accompanied by *TET2* mutations, suggesting that *TET2* and subsequent *RHOA* mutations may pave the way for T-cell transformation [95]. The *RHOA G17V* mouse model has reduced T-cell numbers, but these cells display increased activation upon stimulation and skew toward T_FH_ cell differentiation.

## 5. Current therapies and novel approaches

### 5.1. STAT3/5 *mutations and inhibition*

The STAT protein family is composed of seven members. They share five structural domains: amino-terminal, coiled-coil, DNA-binding, SH_2_, and carboxy‑terminal transactivation/stability domain. The C-terminal domain of STAT3/5 proteins contains two or three amino acid residues that are phosphorylated and crucial for activity, translocation, and gene regulation. Phosphorylation of an essential tyrosine residue promotes parallel dimerization, whereas phosphorylation of serine residues enhances transcriptional elongation and translocation to mitochondria (in the case of STAT3) or the nucleus (in the case of STAT5A).

Normal STAT action is rapid and transient upon response to cytokines/growth factors. Recycling occurs through tyrosine phosphatases and inhibition by degradation is more associated with JAK and receptor proteins. STATs display a tight regulation of the expression of genes whose protein products regulate critical processes such as proliferation, survival, differentiation, senescence, metabolism, angiogenesis, and invasion [123]. Constitutive activation of STAT3/5 is commonly found in MPN and PTCL [20,124]. Consistent with the prediction that oncogenic transcription factors are triggered downstream of many activated drivers, STATs are activated much more commonly than any single genetic driver mutation [125].

Importantly, recurrent somatic *STAT3/5* gain-of-function mutations were found in the SH_2_ domain or their extreme C-terminus [126], acting as driver genes predominantly in PTCL. Understanding how mutations within the JAK-STAT pathway alter chromatin via epigenetic changes is key to gaining insight into reprogrammed gene regulation in cancer to tailor patient-specific therapies.

The final consideration in developing an anticancer therapy concerns the therapeutic index. While a drug may inhibit a pathway critical for cancer cell proliferation or survival, it is equally important that it is not toxic to normal cells. Evidence from experimental systems to human genetic analyses has provided strong support for reasoning that the activity of specific STAT family members can be lost from normal cells without severe consequence, likely due to redundancies in transcriptional regulation under physiologic conditions [127,128]. Taken together, these findings suggest that STATs are valuable targets for cancer therapy.

It has often been argued that STAT transcription factors are not optimal targets for pharmacological inhibition, because their function is not dependent on small surfaces or pockets to which drug-like molecules can bind. However, STATs clearly have discrete domains necessary for their function, including the SH_2_, DNA binding and N-terminal oligomerization domains [129]. These sites can certainly be blocked using a number of strategies that hold promise for therapeutic development.

The first inhibitor of a STAT protein was a peptide molecule [130] and efforts to target STAT signaling for therapeutic purposes are ongoing. To date, inhibition of STAT function has been attempted through several approaches, including N-terminal domain binders [131], oligonucleotides targeting the DNA binding domain [132], and most effectively through use of small molecule compounds that bind the SH_2_ domain to block STAT phosphorylation, dimerization, nuclear transport, and target gene expression [133–135].

The vast majority of medicinal chemistry efforts to target STAT proteins have been conducted to develop specific inhibitors against STAT3 [130–132,136–148], with fewer reports of inhibitory modulators of STAT5A/B. The FDA-approved neuroleptic agent Pimozide was identified in a high-throughput screen as an inhibitor of STAT5 phosphorylation and an inducer of apoptosis in CML cell lines [149]. The underlying mechanism of action is unknown but was suggested to be upstream of STAT5. Furthermore, a non-peptidic chromone-based nicotinyl hydrazine, discovered through a screen of chemical libraries, was shown to weakly inhibit STAT5 activity [150]. This agent selectively inhibited the phosphorylation of STAT5 in lymphoma cell lines by unknown mechanisms. Inhibition of STAT5 activity was also reported for Indirubin derivatives, including E804, which blocked STAT5 phosphorylation and STAT5 DNA-binding activity in CML cells [151], associated with downregulation of *MCL-1* and *BCL2L1* expression. Based on the structure of the compound, the mechanism of inhibition of STAT5 here is most likely suppression of TK activities.

More recently, a number of promising covalent STAT3/5 SH_2_ domain-binding inhibitors have been described [8,133,134,152]. These compounds exhibit potent and selective binding activity for STAT3/5 by effectively disrupting phosphopeptide interactions. The lead agent 13a suppresses STAT3/5 tyrosine phosphorylation and inhibits STAT3/5-mediated gene expression, including downregulation of MYC, Cyclin D1, Cyclin D2, and MCL-1 oncoproteins. Importantly, the dual inhibitory function of STAT3/5 inhibitors is of high clinical relevance since Imatinib-resistant CML cells upregulate and activate STAT3, which represents a major signaling node conferring TKI resistance [8]. Moreover, a feedback upregulation of STAT3 as a common cause of resistance to receptor TK/MEK-targeted therapy was described [153]. Overall, high levels of both STAT3/5 activity are found in most cancer types or stroma cells surrounding MPN or PTCL cells. Taken together, new data and mutational landscape studies provide a rationale for targeting both STAT3 and STAT5 [154]. Furthermore, combining potential STAT3/5 inhibitors with approved TKIs might be beneficial in treating cancer. STAT dimerization and signaling can also be blocked by inhibiting upstream JAK kinases, which we discuss next.

### 5.2. JAK kinase inhibitors

Ruxolitinib partially inhibits the activity of JAK1/2 and is the first drug approved by the FDA for MPN patients. It is prescribed as a targeted therapy for treatment of patients with primary MF, PV, and ET [155]. During clinical trials, it was shown to reduce spleen size, abdominal discomfort, bone pain, night sweats, and itching, as well as diminish the level of inflammatory cytokines in MPN patients.

A number of other drugs that inhibit JAK kinases are currently in clinical trials, including Pacritinib, Momelotinib and NS-018. Pacritinib, a dual JAK2 and FLT3 TKI, is being compared with best available therapy in Phase III trials in patients with MF [156]. Momelotinib performed better than Ruxolitinib with respect to anemia-related end points, but formal statistical testing was not undertaken. How Momelotinib might improve anemia despite inhibiting JAK1/2 is not well understood, but one putative mechanism could be the inhibition of ALK2-mediated hepcidin expression in the liver, which in turn results in increased release of storage iron and promotion of erythropoiesis. NS-018 is a JAK2-selective inhibitor with an IC_50_ of <1 nM and it has 30- to 50-fold greater selectivity for JAK2 than for other JAK family kinases (JAK1, JAK3, TYK2), and can also inhibit SRC-family kinases. NS-018 potently decreases viability of cell lines expressing constitutively activated JAK2, suppresses endogenous erythroid colony formation by primary cells from PV patients, reduces leukocytosis and splenomegaly, improves BM fibrosis, and prolongs survival in a mouse model of JAK2 V617F-driven MF without causing peripheral anemia or thrombocytopenia [157]. Still, Ruxolitinib remains superior in clinical use and will be challenging to improve upon.

### 5.3. mTOR inhibitors

Everolimus (also known as RAD001) is a broadly used inhibitor of the mTOR/AKT pathway, which is commonly upregulated in MF hematopoietic cells and appears to contribute to abnormal cell growth. Everolimus was well tolerated in phase I and II clinical trials and was able to reduce both spleen size and systemic symptoms. However, no major sustained responses were seen in these patients [158].

### 5.4. Epigenetic drugs

Epigenetic drugs change the way genes are organized to make them more or less accessible for use by the cell. Studies have found that Givinostat (HDAC inhibitor) and two hypomethylating drugs, Azacitidine and Decitabine, were minimally effective in treating MF, in contrast to their effectiveness in treating PV. Another HDAC inhibitor, Panobinostat, is currently under investigation. HDAC inhibitors are pleiotropic agents that have multiple potential mechanisms of action in MPN cells, prominent among them being downregulation of JAK2 via inhibition of the chaperone protein function of HSP90. Givinostat and Vorinostat are clearly active in patients with PV and ET, producing both spleen and hematologic responses in a substantial proportion of patients, apparently without regard to the mutational status of JAK2.

Vorinostat, a class I and II HDAC inhibitor, was approved more than 10 years ago for the treatment of CTCL [159]. Romidepsin, a pan-HDAC inhibitor, is also approved for use in CTCL patients as well as for relapsed and refractory PTCL. A third inhibitor, the pan-HDAC inhibitor Belinostat, was also more recently approved for relapsed and refractory PTCL cases [160,161]. The overall responses were 25% for Romidepsin and 26% for Belinostat [162,163]. Additional indications for Romidepsin are currently being evaluated as a combinatorial treatment, for instance, with Bortezomib, Carfilzomib (both proteasome inhibitors), 5-Azacytidine, or CHOP. Chidamide, as well as acting as an HDAC inhibitor, is so far only approved for PTCL treatment in China and used as a monotherapy or in combination with chemotherapy [164,165].

Because JAK2 interacts with the chaperone HSP90, pharmacologic inhibition of HSP90 was proposed to cause misfolding and degradation of JAK2. This was shown in MPN cell lines, primary MPN patient samples, and mouse models of PV and ET treated with the HSP90 inhibitor PU-H71, without degradation of JAK2 in normal tissues or substantial toxicity. Degradation of JAK2 via HSP90 inhibition has also been shown to be a way of circumventing persistent signaling with JAK2 inhibition. Synergism between the HSP90 inhibitor AUY922 and the JAK2 inhibitor TG101209 was demonstrated in human CD34^+^ MPN cells, which exhibited significantly greater apoptosis than did normal hematopoietic progenitor cells. Combination therapy with PU-H71 and Ruxolitinib was shown to be more potent in inhibiting JAK2 downstream signaling than Ruxolitinib alone [166]. This translated to improvements in blood counts, spleen weights, and BM fibrosis in transgenic mice. The combination of Ruxolitinib and Decitabine appears promising in patients with accelerated or blast phase MPN (post-MPN acute myeloid leukemia) in small studies [167].

### 5.5. TP53 reactivation and TP53* targeting

Rescuing unstable TP53 protein pools upon hotspot mutation demonstrates feasibility to target transcription factor function. This is independent of whether *TP53* was mutated or aberrantly activated due to upstream mutations in negative-regulators. PRIMA-1 and its derivative PRIMA-1^MET^ (also called APR-246) can restore wild-type protein conformation to TP53*. This restores transcriptional activity of normal TP53 that senses DNA damage, leading to expression of *PUMA, NOXA*, and *BAX* in *TP53*-mutated cancer cells [168,169]. PRIMA-1 compounds are converted intracellularly to the Michael acceptor methylene quinuclidinone, subsequently binding covalently to cysteines of TP53*. It will be important in a clinical setting to tailor the strategy to specific MPN or PTCL subtypes dependent on the *TP53* mutational status. Furthermore, MDM2 and MDMX expression levels are of relevance, which we discuss next.

MDM2 is an important negative regulator of TP53, and small-molecule inhibitors of MDM2 can trigger apoptosis in cells with intact TP53 function through TP53-activation. Because type I interferons (IFN) target JAK2 V617F^+^ progenitors in PV through activation of MAPK and STAT1, thereby increasing *TP53* transcription, the combination of IFN with MDM2 inhibitors, which prevent the degradation of TP53, provides an opportunity to induce TP53-dependent apoptosis [170]. Indeed, combination treatment with IFN and the MDM2 antagonist Nutlin-3 triggered apoptosis in PV CD34^+^ cells and inhibited proliferation of these cells to a greater extent than normal CD34^+^ cells [170]. The combination also reduced the proportion of JAK2 V617F progenitors in PV patients. Combination treatment of PV and primary MF CD34^+^ cells, followed by transplantation into immunodeficient mice, decreased the extent of donor-derived chimerism as well as the *JAK2 V617F* allele burden, suggesting that such combinatorial approaches may deplete MPN hematopoietic stem cells [170]. The clinical candidate MDM2 antagonist Idasanutlin is currently in a phase I trial in patients with PV or ET, with a provision for adding pegylated IFN in subjects without or with partial remission after three cycles of therapy.

An overview of the drugs currently undergoing clinical trials for MPN and PTCL is displayed in Table 3.

**Table 3.** List of drugs and their targets currently undergoing clinical trials for MPN and/or PTCL disease as mono- and/or combination therapies as of October 2017 (*https://clinicaltrials.gov/*). Drugs that are involved in clinical trials for both MPN and PTCL are highlighted in *italic*. Only targeted therapy drugs are listed (no chemotherapy or immunotherapy drugs included).

MPN		PTCL	
Drug	Target	Drug	Target
*Ruxolitinib*	JAK1/2	*Ruxolitinib*	JAK1/2
Momelotinib		Cerdulatinib	SYK/JAK
Itacitinib	JAK1	ASN002	
NS-018	JAK2	Everolimus	mTOR/AKT pathway
Pacritinib		Temsirolimus	
LY2784544		BMS-906024	Notch
*Umbralisib (TGR-1202)*	PI3Kδ	LY3039478	
Idelalisib		Tipifarnib	Ras (posttranslational modification)
INCB050465		*Umbralisib (TGR-1202)*	PI3Kδ
Rigosertib	PI3K and PLK pathways	CPI-618	α-ketoglutarate dehydrogenase
Glasdegib (PF-04449913)	Sonic hedgehog pathway	Alisertib	Aurora A kinase
Sonidegib (LDE225)		DS-3201b	EZH2
*LCL161*	cIAP1 and cIAP2	*Decitabine*	Hypomethylation
Idasanutlin (RG7388)	TP53–MDM2	*Panobinostat*	Pan-HDAC
PRIMA-1^MET^ (APR-246)	TP53	Romidepsin	
*IMG-7289*	*LSD1*	Belinostat	
Givinostat (ITF2357)	Class I and class II HDACs	AR-42	
*Panobinostat*	Pan-HDAC	Chidamide	
Azacitidine	Hypomethylation	Bortezomib	Proteasome
*Decitabine*		Carfilzomib	
PU-H71	HSP90	Ixazomib (MLN 9708)	
AUY922			

## 6. Expert opinion

Small molecule inhibitors targeting key drivers in MPN or PTCL hold the greatest promise to reach the clinic. Their size, polarity, solubility, pharmacokinetics and pharmacodynamics, and toxic side-effects can be improved through medicinal chemistry approaches, and structural modeling based on lead compounds could improve targeting efficacy. We need to better understand and map how mutated disease drivers such as epigenetic remodelers, *JAK-STAT* gain-of-function mutations, TP53*, and hyperactive GTPases interact and cooperate in specific cell types. In summary, structural modeling and protein interaction studies can reveal a detailed, atomic-level understanding of vulnerable nodes. During therapy with targeted drugs, new subclones with other driver mutations may escape and lead to relapses, which points to the need to develop new drugs with broader multi-target activities. Furthermore, *in vivo* targeting of suitable animal models, accurate biological read out systems with the right combination of driver mutations, and related early phase clinical trials focused on specific patient subgroups could increase the repertoire of therapeutic approaches to target MPN and PTCL. We stand at a crossroads in understanding key drivers in cancer biology, but we still need to understand how they cooperate to manifest into neoplasia. How interactions between the DNA damage and checkpoint control machinery, or epigenetic gene regulation influences or connects to JAK-STAT driver mutations is still under investigation. Hematologic cancer research defines MPN and PTCL as different disease entities; however, insights into epigenetics and mutational landscapes with expression profiling point to more similarity between the two diseases, suggesting the potential for common targeting strategies.

## Article highlights

- MPN and PTCL are both aggressive hematopoietic malignancies, requiring targeted treatment.
- High occurrence of resistance mechanisms in MPN patients limits effective use of Ruxolitinib in clinics.
- Treatment options for PTCL patients are limited to chemotherapy due to a lack of potential molecular targets for this disease.
- High mutational rates in epigenetic regulators, as well as other common signaling pathways, have been reported in both MPN and PTCL.
- Potential strategies to improve treatment of both MPN and PTCL patients include drugs targeting these commonly mutated pathways.
